# Association between weight change and the predicted 10-year risk for atherosclerosis cardiovascular disease among U.S. older adults: data from National Health and Nutrition Examination Survey 1999–2018

**DOI:** 10.3389/fpubh.2023.1183200

**Published:** 2023-10-16

**Authors:** Yuxuan Peng, Hongzheng Li, Feifei Liao, Jieming Lu, Wenwen Yang, Ling Tan, Aimei Lu, Yue Wei, Linzi Long, Hua Qu, Changgeng Fu

**Affiliations:** ^1^Xiyuan Hospital of China Academy of Chinese Medical Sciences, Beijing, China; ^2^National Clinical Research Center for Cardiovascular Diseases of Traditional Chinese Medicine, Beijing, China; ^3^Beijing University of Chinese Medicine, Beijing, China

**Keywords:** weight change, cardiovascular disease, NHANES, 10-year ASCVD risk, older adults

## Abstract

**Background:**

It remains controversial regarding the association between weight change and cardiovascular disease risk in older adults (aged ≥60 years). This study aimed to evaluate the association between weight change and the predicted 10-year atherosclerotic cardiovascular disease (ASCVD) risks in older adults.

**Methods:**

This study used data from the National Health and Nutrition Examination Survey (NHANES). Older adults aged 60–79 years who were free of self-reported ASCVD at the time of the NHANES interview were included. Data were collected from January 1999 to December 2018 and analyzed in March 2022. We focused on the associations between weight change and the 10-year ASCVD risks with the percentage change in weight during short-term (1 year) and long-term (10 years), which categorized as moderate to high weight loss (≥10%), small weight loss (5.1–9.9%), stable weight (±5%), small weight gain (5.1–9.9%), and moderate to high weight gain (≥10%).

**Results:**

The number of participants was 1,867 (mean age 67.49 years; 42.10% female) for the long-term interval (10 years) in our analysis, and 1894 for the short-term interval (1 years). We only observed an inverse association between long-term weight loss and the 10-year ASCVD risk in fully adjusted model (loss ≥ 10%: *β* = 2.52, 95%CI = 0.98, 4.05; loss 5.1% ~ 9.9%: *β* = 2.99, 95% CI = 1.30, 4.68), but all intervals of weight gain ≥5% were not significant associated with higher risk than stable weight. However, in the subgroup analyses, the association between long-term weight loss and the 10-year ASCVD risk was not significant in old-old (aged 75–79), obesity (BMI ≥ 35 kg/m^2^), intentional weight loss, moderate physical activity and diabetics.

**Conclusion:**

Older adults (aged 60–79 years) with weight loss >5% over the past 10 years have excess predicted 10-year ASCVD risk. Our study supports the benefits of stable weight in promoting cardiovascular health in older adults.

## 1. Introduction

The increasing incidence of overweight and obesity among the aging population is a growing public health problem worldwide (1). Between 2007 and 2016, the proportion of obesity increased from 35.1 to 41.0% in older Americans, giving rise to a significant future burden on the U.S. healthcare systems (2). Substantial epidemiologic evidence indicates that excess body weight is associated with a higher risk of mortality, primarily due to atherosclerotic cardiovascular disease (ASCVD) (3, 4). It is not unexpected since obesity is clearly associated with most of the classical cardiovascular risk factors like hypertension, hyperlipidemia, and diabetes (5). However, whether losing weight could have a favor effect on decreasing cardiovascular events risk in older adults remains controversial.

Previous studies on the association between weight loss and long-term cardiovascular outcomes in older adults are limited. Although several studies have indicated that weight loss improved physical function and reduced frailty in obese older adults (6, 7). Increasing evidence suggested that weight loss was not uniformly associated with improved long-term survival (8). A meta-analysis reported that weight loss and weight gain were associated with a 59 and 10% increased risk of mortality respectively, suggesting an obesity paradox in older adults (9). In addition, the effect of weight loss among cardiovascular disease patients is also controversial (10). A meta-analysis of 35,335 patients (mean age 64 years) showed that, overall, weight loss was associated with a higher risk of cardiovascular events, but intentional weight loss was associated with improved outcomes (11). However, it remains unknown whether weight loss is associated with an increased risk of ASCVD events among older adults who are free of a prior heart attack or stroke. It is imperative to understand the health impact of long-term weight change on ASCVD risk in the general older population.

Therefore, the primary goal of this study was to examine the association between weight change and the 10-year predicted ASCVD risk in older U.S. adults (aged ≥60 years) using data from the 1999–2018 National Health and Nutrition Examination Survey (NHANES).

## 2. Methods

### 2.1. Database and study subjects

In this study, the data were obtained from the NHANES (1999–2018). This is an ongoing cross-sectional survey conducted by the National Center for Health Statistics (NCHS), designed to be representative of the U.S. non-institutionalized, civilian population. During a home interview, data are collected on demographic, socioeconomic, and health-related topics (including weight history). A separate examination collects standardized physical assessments and laboratory measurements. The survey obtained written informed consents from all participants prior to data collection. Methodological details about the NHANES are available at: www.cdc.gov/nchs/nhanes/.

**Figure 1:**
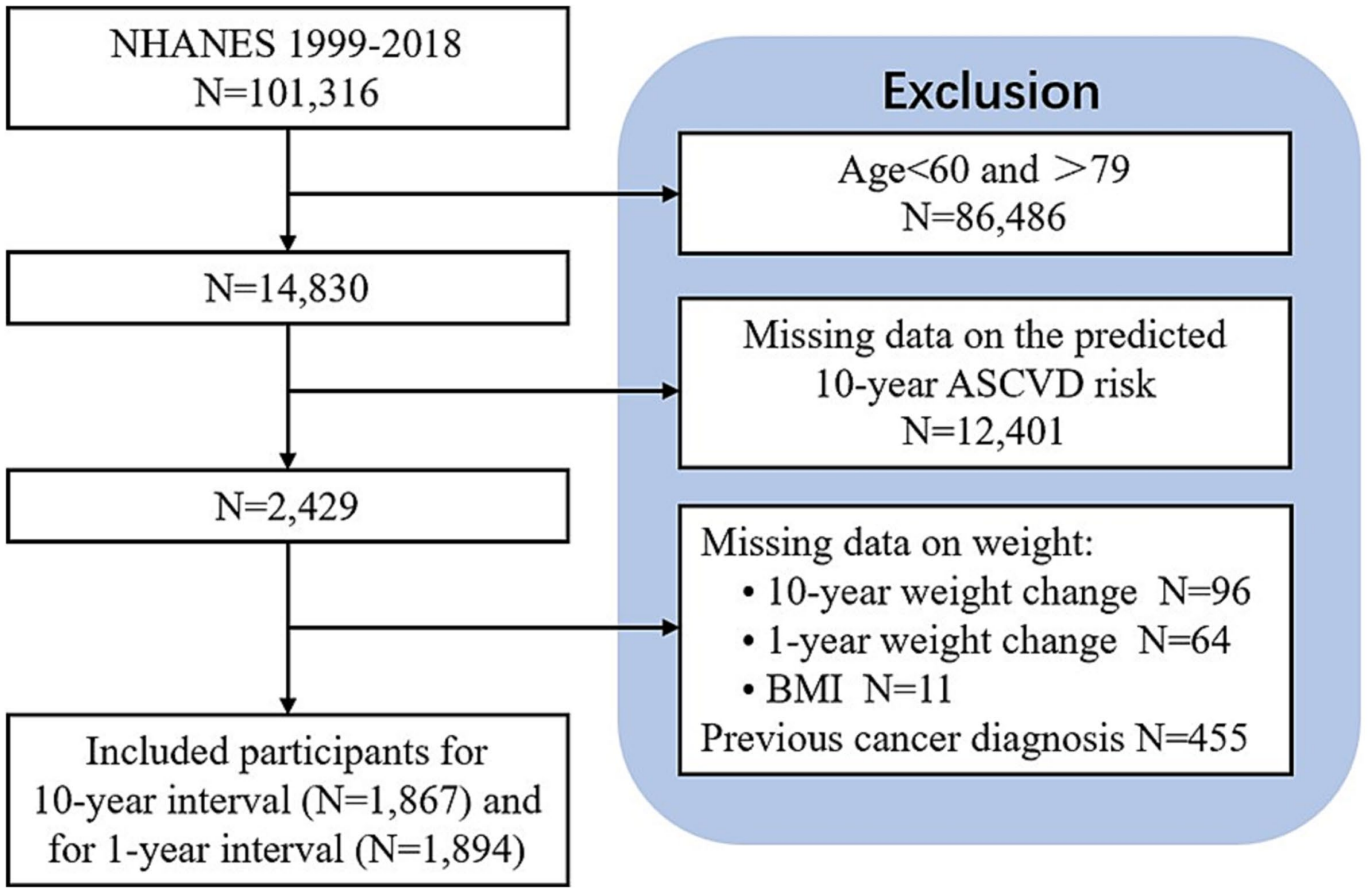
Flowchart of the study population.

Older adults were defined as those of age 60 years and over (12). To estimate the 10-year ASCVD risk, the analytic sample was limited to 2,429 participants aged 60–79 years who were free of self-reported ASCVD at the beginning of the survey and met with high-density lipoprotein cholesterol (HDL-C) 20–100 mg/dL, total cholesterol (TC) 130–320 mg/dL, diastolic BP 30–140 mmHg and systolic BP 90–200 mmHg. After the exclusion of the participants with missing data on 10-year weight change (*n* = 96), 1-year weight change (*n* = 64) and BMI (*n* = 11), and those with a previous cancer diagnosis (*n* = 455), the number of participants included in the analysis was 1,867 for the long-term interval (10 years) and 1,894 for the short-term interval (1 years) (Figure 1).

### 2.2. Assessment of the predicted 10-year ASCVD risk

The primary outcome of this study was the 10-year ASCVD risk, defined as a first non-fatal myocardial infarction (MI), coronary heart disease (CHD) death, or fatal or non-fatal stroke over a 10-year period (13). According to the new pooled cohort equations (PCEs) introduced by the American College of Cardiology (ACC) and the American Heart Association (AHA) in 2013, the predictors used in estimating the 10-year risk of a first ASCVD event included age, sex, race, TC, HDL-C, treatment for hypertension, diabetes and current smoking status (13).

Data on age, sex, and race/ethnicity was obtained from the demographic questionnaire. The amount of TC (mg/dL) and HDL (mg/dL) was obtained from the laboratory file. Blood pressure was each calculated as the average of three readings. Participants self-reported currently smoking (yes, no), taking any blood pressure medications (yes, no), and had ever been told they had diabetes by a medical doctor (yes, no). Histories of diseases including CHD, MI, stroke, diabetes and cancer were ascertained through the question “Has a doctor or other health professional told you that you had diseases?”

### 2.3. Assessment of weight change

We used long-term (10 years) and short-term (1 year) weight change indicators according to the interval over which the change was assessed. The percentage change in weight was calculated from the difference between the present weight and the past weight, the specific formula was shown as followed:


$$\frac{W-W_{0}}{W_{0}}\times 100\%\text{,}$$

where W refers to the present weight and W_0_ refers to their previous weight. All participants were asked to recall the weight 10 years ago or 1 year ago, and measured current weight in the NHANES examination. We classified the weight change into five categories: moderate-to-large weight loss (≥ 10%), small weight loss (5.1–9.9%), stable weight (± 5%), small weight gain (5.1–9.9%), and moderate-to-large weight gain (≥ 10%).

### 2.4. Assessment of covariates

The following covariates were included: age, sex, race, Body Mass Index (BMI), marital status, educational level, family income-to-poverty ratio, and physical activity. Marital status was categorized as married/partnered (married and living as married) and single/no partner (widowed, divorced, separated and never married). Educational level was classified as lower category (less than high school), intermediate category (high school graduate/GED, some college or AA degree) and higher category (college graduate or above). Physical activity was defined as sports, fitness and recreational activities, excluding work and transport activities. Physical activity was categorized as vigorous activity (cause large increases in breathing or heart rate), moderate activities (cause a small increase in breathing or heart rate) and none.

### 2.5. Statistical analysis

Categorical variables were expressed as frequency, and continuous variables were expressed as means ± standard deviations. Demographic and clinical data between the weight change groups were compared using the Kruskal-Wallis test and Fisher’s exact test. Multiple linear regression analyses were used to estimate the independent relationship between weight change and the predicted 10-year risk of ASCVD events. When calculating the relative ASCVD risk of each weight change category, we used the “stable weight” category as the reference. In addition to the relative 10-year ASCVD risk of each weight change category, we fit a smoothing spline curve to examine the non-linearity of weight change and the 10-year ASCVD risks. Moreover, we performed stratified analyses by age, sex, race/ethnicity, current BMI, intention to lose weight, physical activity, treatment for hypertension, diabetes and current smoking status.

Three models were constructed: Model 1 was adjusted for none; Model 2 was adjusted for sex, age, and race/ethnicity; Model 3 was adjusted for sex, age, race/ethnicity, body mass index, income-poverty ratio, physical activity, education level, and marital status. In the subgroup analysis, the model is not adjusted for the stratification variable itself.

We used multiple imputations, based on 5 replications and a chained equation approach method in the RMI procedure, to account for missing data. All statistical analyses were performed by using R version 3.4.3 (The R Foundation)^1^ and EmpowerStats software (X&Y solutions, Inc., Boston, MA)^2^ and Graphpad Prisma 8.3.0, with statistically significant set at *p* < 0.05.

## 3. Results

### 3.1. Characteristics

The description of sociodemographic and medical characteristics of the participants with long-term (10 years) weight change was presented in Table 1. A total of 1,867 older adults aged 60–79 years were enrolled in our analysis. Of all these participants, the mean age was 67.47 years (SD = 5.35), and 42.10% were female. For all participants, the mean predicted 10-year ASCVD risk was 23.21 (SD = 13.15). The mean BMI was 30.23 kg/m^2^ (SD = 6.21). The distribution of 10-year weight change was 27.05% for weight stable (± 5.0%), 13.87% for moderate-to-large weight loss (> − 10%), 10.18% for small weight loss (−5.1 to −9.9%), 13.93% for small weight gain (5.1~9.9%) and 34.98% for moderate-to-large weight gain (>10%). Among different groups of weight change, age, sex, race/ethnicity, BMI, income-poverty ratio, educational level, marital status, physical activity, smoker, diabetes, DBP, TC, and HDL were all significantly different (*p* < 0.05). Overall, older adults who had weight loss were more likely to be a racial/ethnic minority, single/no partner, and had lower income-to-poverty ratios and educational attainment, when compared to those with a stable weight. The descriptive statistics for the participants with short-term (1 year) weight change are shown in Supplementary material 1.

**Table 1:** Characteristics of study participants according to 10-year weight change patterns in National Health and Nutrition Examination survey, 1999–2018.

Weight change	All participants (*n* = 1,867)	Weight stable (*n* = 505)	Moderate-to-large weight loss (*n* = 259)	Small weight loss (*n* = 190)	Small weight gain (*n* = 260)	Moderate-to-large weight gain (*n* = 653)	*p*-value
Age (years), mean (SD)	67.47 ± 5.35	67.98 ± 5.38	68.22 ± 5.29	68.56 ± 5.52	66.72 ± 5.23	66.76 ± 5.24	**<0.001**
Sex, n (%)							**<0.001**
Male	1,081 (57.90%)	334 (66.14%)	150 (57.92%)	129 (67.89%)	169 (65.00%)	299 (45.79%)	
Female	786 (42.10%)	171 (33.86%)	109 (42.08%)	61 (32.11%)	91 (35.00%)	354 (54.21%)	
Race/ethnicity, n (%)							**0.002**
Mexican American	287 (15.37%)	73 (14.46%)	49 (18.92%)	38 (20.00%)	40 (15.38%)	87 (13.32%)	
Other Hispanic	153 (8.19%)	35 (6.93%)	23 (8.88%)	16 (8.42%)	16 (6.15%)	63 (9.65%)	
Non-Hispanic white	814 (43.60%)	242 (47.92%)	95 (36.68%)	63 (33.16%)	131 (50.38%)	283 (43.34%)	
Non-Hispanic Black	529 (28.33%)	125 (24.75%)	83 (32.05%)	61 (32.11%)	68 (26.15%)	192 (29.40%)	
Other race	84 (4.50%)	30 (5.94%)	9 (3.47%)	12 (6.32%)	5 (1.92%)	28 (4.29%)	
BMI (kg/m^2^), mean ± SD	30.23 ± 6.21	28.42 ± 5.21	27.32 ± 5.60	27.60 ± 5.51	30.69 ± 4.85	33.37 ± 6.40	**<0.001**
Income poverty ratio, mean ± SD	2.52 ± 1.57	2.70 ± 1.61	1.98 ± 1.38	2.50 ± 1.54	2.89 ± 1.60	2.45 ± 1.56	**<0.001**
Educational level, n (%)							**<0.001**
Lower	624 (33.46%)	157 (31.09%)	129 (50.00%)	64 (33.68%)	63 (24.23%)	211 (32.36%)	
Intermediate	926 (49.65%)	251 (49.70%)	99 (38.37%)	99 (52.11%)	147 (56.54%)	330 (50.61%)	
Higher	315 (16.89%)	97 (19.21%)	30 (11.63%)	27 (14.21%)	50 (19.23%)	111 (17.02%)	
Marital status, n (%)							**0.006**
Married/partnered	1,143 (61.65%)	333 (66.60%)	144 (56.03%)	108 (57.14%)	173 (66.80%)	385 (59.32%)	
Single/no partner	711 (38.35%)	167 (33.40%)	113 (43.97%)	81 (42.86%)	86 (33.20%)	264 (40.68%)	
Physical activity, n (%)							**0.017**
Vigorous activity	204 (10.93%)	75 (14.85%)	25 (9.65%)	19 (10.00%)	27 (10.38%)	58 (8.88%)	
Moderate activity	553 (29.62%)	158 (31.29%)	64 (24.71%)	56 (29.47%)	84 (32.31%)	191 (29.25%)	
No	1,110 (59.45%)	272 (53.86%)	170 (65.64%)	115 (60.53%)	149 (57.31%)	404 (61.87%)	
Smoker, n (%)							**<0.001**
Yes	482 (25.82%)	104 (20.59%)	107 (41.31%)	59 (31.05%)	50 (19.23%)	162 (24.81%)	
No	1,385 (74.18%)	401 (79.41%)	152 (58.69%)	131 (68.95%)	210 (80.77%)	491 (75.19%)	
Diabetes, n (%)							**<0.001**
Yes	517 (27.69%)	118 (23.37%)	103 (39.77%)	65 (34.21%)	69 (26.54%)	162 (24.81%)	
No	1,350 (72.31%)	387 (76.63%)	156 (60.23%)	125 (65.79%)	191 (73.46%)	491 (75.19%)	
Treatment for hypertension, n (%)							0.051
Yes	1710 (91.59%)	457 (90.50%)	247 (95.37%)	173 (91.05%)	230 (88.46%)	603 (92.34%)	
No	157 (8.41%)	48 (9.50%)	12 (4.63%)	17 (8.95%)	30 (11.54%)	50 (7.66%)	
SBP (mmHg), mean ± SD	136.19 ± 19.55	135.66 ± 19.23	136.13 ± 21.90	139.06 ± 20.31	134.68 ± 18.19	136.38 ± 19.08	0.193
DBP (mmHg), mean ± SD	70.53 ± 12.28	70.38 ± 12.03	68.43 ± 12.05	71.75 ± 12.93	70.73 ± 11.83	71.05 ± 12.46	**0.029**
TC (mg/dL), mean ± SD	196.11 ± 36.68	196.99 ± 35.87	190.37 ± 36.16	195.02 ± 37.89	197.02 ± 36.21	197.67 ± 37.22	0.087
HDL (mg/dL), mean ± SD	67.47 ± 5.35	53.04 ± 15.72	55.15 ± 15.70	53.16 ± 15.81	51.15 ± 14.48	51.33 ± 14.36	**0.005**
The 10-year ASCVD risk, mean ± SD	23.21 ± 13.15	23.22 ± 13.16	26.59 ± 14.17	27.84 ± 13.44	21.69 ± 12.07	21.13 ± 12.46	**<0.001**

Boldface indicates statistical significance (*p* < 0.05). BMI, body mass index; SBP, systolic blood pressure; DBP, diastolic blood pressure; TC, total cholesterol; HDL-C, high-density lipoprotein cholesterol; ASCVD, atherosclerotic cardiovascular disease; SD, standard deviation.

### 3.2. 10-year weight change and the predicted risk of ASCVD events

**Figure 2:**
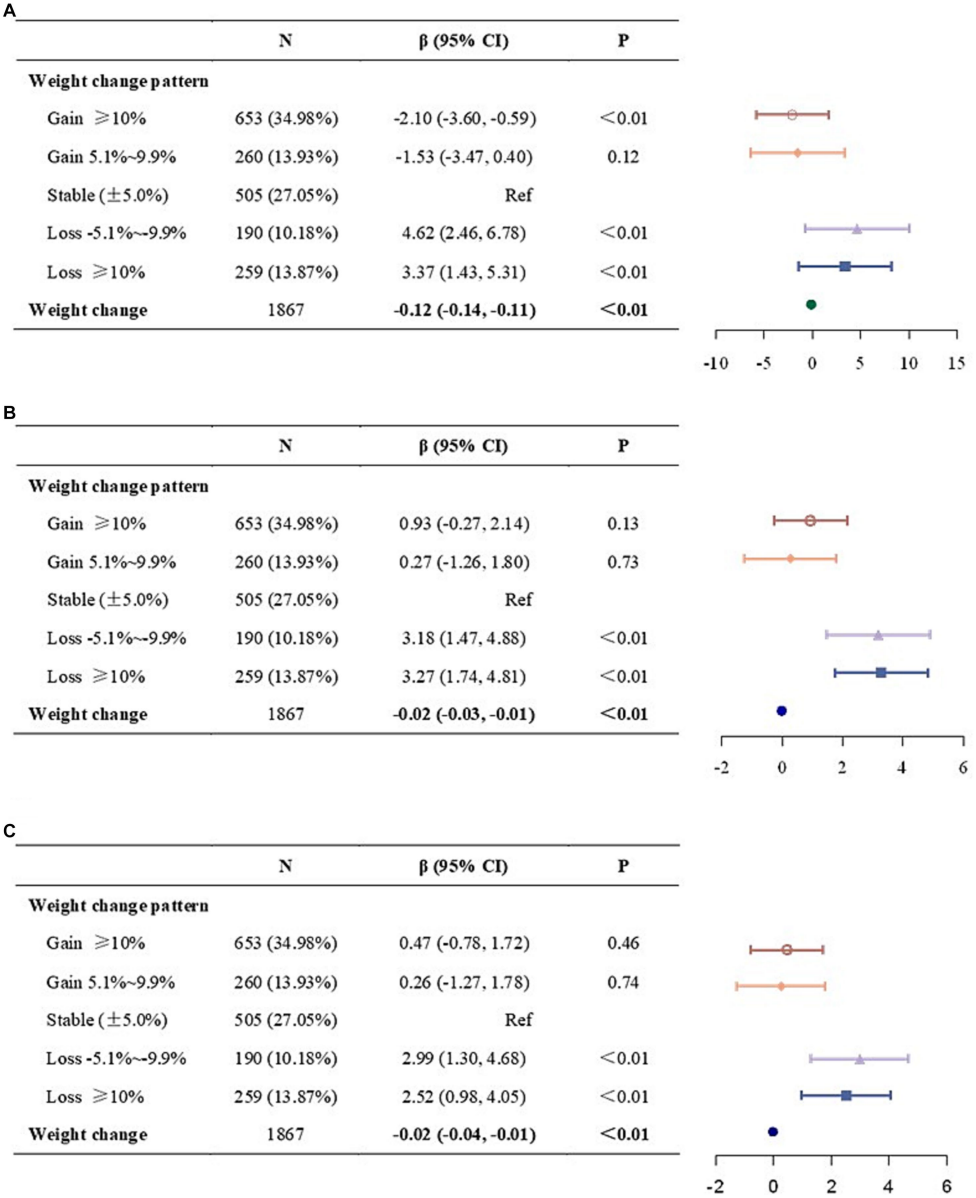
Association between 10-year weight change and the 10-year ASCVD risk. **(A)** Model 1, no covariates were adjusted. **(B)** Model 2, age, sex, and race/ethnicity were adjusted. **(C)** Model 3, age, sex, race/ethnicity, body mass index, income-poverty ratio, physical activity, education level, and marital status were adjusted. ASCVD, atherosclerotic cardiovascular disease; CI, confidence interval.

Results of multiple linear regression analyses of 10-year weight change and the 10-year risk of ASCVD events were displayed in Figure 2. Non-adjusted was displayed in Model 1 (Figure 2A), adjusted for age, sex, and race/ethnicity was displayed in Model 2 (Figure 2B), and adjusted for Model 2 plus BMI, income-poverty ratio, physical activity, education level, and marital status was displayed in Model 3 (Figure 2C). We observed a significant inverse association between weight change and the 10-year ASCVD risk.

Similar results were found when weight change was divided into five categories. Compared to weight stable (± 5.0%), both moderate-to-large (≥ 10%) and small (−5.1% ~ −9.9%) weight loss was associated with a higher risk in Model 1 and Model 2. In fully adjusted model (Model 3), the significant association remained unchanged (loss≥10%: *β* = 2.52, 95%CI = 0.98, 4.05; loss 5.1% ~ 9.9%: *β* = 2.99, 95% CI = 1.30, 4.68). While neither Model 2 nor Model 3 found no significant association between the weight gain categories and the 10-year ASCVD risk, weight gain ≥10% was associated with lower risk in non-adjusted model (*β* = −2.10, 95% CI = −3.60, −0.59).

**Figure 3:**
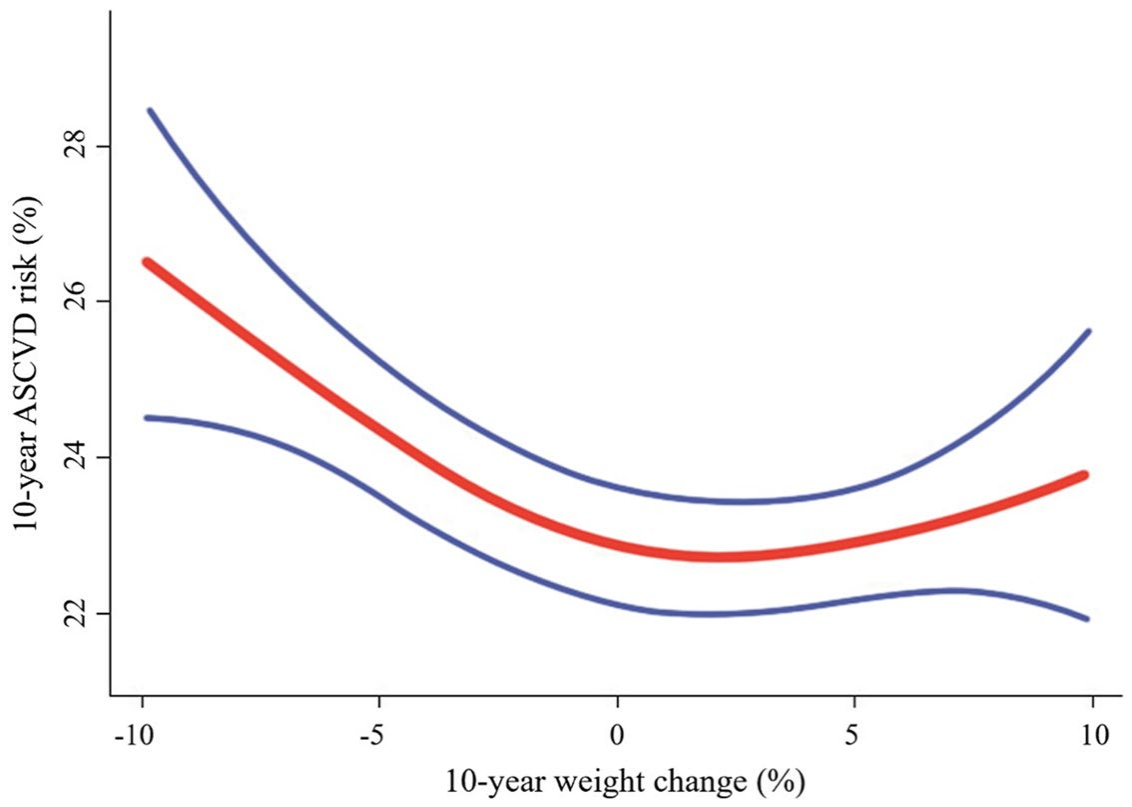
Correlation between 10-year weight change (−9.9 to 9.9%) and the 10-year ASCVD risk. Solid red line represents the smooth curve fit between variables. The area between two blue lined is expressed as a 95% CI. Age, sex, race/ethnicity, body mass index, income poverty ratio, physical activity, education level, and marital status were adjusted. ASCVD, atherosclerotic cardiovascular disease.

To check the dose–response relationship between continuous 10-year weight change (−9.9 to 9.9%) and the predicted 10-year risk of ASCVD events, we plotted the spline curves, as seen in Figure 3. This result confirmed the dose–response relationship was most pronounced in the link between weight loss and the 10-year ASCVD risk: greater weight loss was associated with higher 10-year ASCVD risk.

However, this was not the case for short-term weight change. Each category had no significant association with 10-year ASCVD risk, as seen in Supplementary material 2.

Table 2 presents the results stratified according to age, sex, race/ethnicity, BMI, physical activity, Intention to lose weight, treatment for hypertension, diabetes, and current smoking status. Compared to people with stable weight, it can be observed that people who lose weight may associate with a higher 10-year risk, no matter with ages (except for people aged ≥75 years) and sexes in Model 3. However, in the subgroup analyses, the association between weight loss and the 10-year ASCVD risk was non-significant in older adults who were obese (≥ 35 kg/m^2^), had intentional weight loss, and had moderate physical activity. In addition, moderate physical activity significantly decreases the 10-year ASCVD risk in older adults with moderate-to-large weight gain (*β* = −2.70, 95% CI = −4.97, −0.43). We also found that for diabetics, long-term weight change showed no significant association with the 10-year ASCVD risk. Subgroup analyses of short-term weight change and the 10-year ASCVD risk are presented in Supplementary material 3.

**Table 2:** Stratified analyses of the association between 10-year weight change and the 10-year ASCVD risk.

	Weight stable	Moderate-to-large weight loss	Small weight loss	Small weight gain	Moderate-to-large weight gain
Age					
60–64	Ref	**2.55 (0.29, 4.80)**	1.61 (−0.95, 4.17)	−0.84 (−2.86, 1.17)	−0.69 (−2.40, 1.03)
65–69	Ref	**4.45 (1.70, 7.20)**	**4.12 (1.15, 7.10)**	−0.59 (−3.35, 2.17)	0.52 (−1.77, 2.81)
70–74	Ref	1.26 (−2.04, 4.55)	**5.50 (1.89, 9.11)**	0.40 (−2.99, 3.79)	2.07 (−0.70, 4.84)
75–79	Ref	0.57 (−4.86, 6.00)	−1.21 (−6.9, 4.47)	3.30 (−3.06, 9.66)	1.95 (−2.84, 6.73)
Sex					
Male	Ref	**2.43 (0.41, 4.44)**	1.76 (−0.35, 3.87)	0.29 (−1.65, 2.22)	−0.17 (−1.85, 1.51)
Female	Ref	**2.99 (0.58, 5.40)**	**5.81 (2.93, 8.68)**	−0.07 (−2.58, 2.44)	1.47 (−0.44, 3.38)
Race/Ethnicity					
Mexican American	Ref	2.51 (−0.72, 5.75)	2.40 (−1.06, 5.86)	1.31 (−2.1, 4.72)	−0.58 (−3.44, 2.28)
Other Hispanic	Ref	**6.24 (1.2, 11.29)**	**9.12 (3.61, 14.62)**	1.68 (−3.97, 7.33)	2.11 (−2.16, 6.39)
Non-Hispanic white	Ref	2.08 (−0.05, 4.21)	1.71 (−0.77, 4.18)	0.23 (−1.69, 2.14)	0.46 (−1.19, 2.11)
Non-Hispanic Black	Ref	2.82 (−0.57, 6.22)	3.31 (−0.37, 7.00)	0.00 (−3.58, 3.58)	1.65 (−1.23, 4.52)
Other race	Ref	2.42 (−5.61, 10.45)	−1.33 (−8.36, 5.70)	−3.68 (−14.19, 6.83)	−3.99 (−9.78, 1.80)
BMI					
<25.0	Ref	**3.53 (0.11, 6.95)**	1.23 (−2.48, 4.94)	1.86 (−3.71, 7.42)	−0.45 (−5.38, 4.47)
25.0–29.9	Ref	1.53 (−0.82, 3.88)	**3.56 (1.03, 6.09)**	0.67 (−1.66, 3.00)	−0.53 (−2.45, 1.40)
30.0–34.9	Ref	1.46 (−1.82, 4.75)	**4.36 (0.67, 8.05)**	−1.07 (−3.72, 1.58)	−0.13 (−2.47, 2.21)
≥35.0	Ref	2.43 (−2.47, 7.34)	1.54 (−4.07, 7.15)	1.72 (−2.3, 5.75)	2.54 (−0.49, 5.57)
Physical activity					
Vigorous activity	Ref	3.95 (−0.20, 8.10)	**5.22 (0.69, 9.75)**	0.77 (−3.23, 4.77)	−0.23 (−3.57, 3.12)
Moderate activity	Ref	0.46 (−2.37, 3.30)	1.60 (−1.34, 4.54)	−2.14 (−4.73, 0.45)	**−2.70 (−4.97,-0.43)**
No	Ref	**3.31 (1.25, 5.37)**	**3.46 (1.15, 5.78)**	1.49 (−0.64, 3.61)	**1.98 (0.28, 3.69)**
Intention to lose weight					
Yes	Ref	1.38 (−0.96, 3.72)	1.39 (−1.39, 4.18)	−0.34 (−2.45, 1.77)	0.29 (−1.43, 2.02)
No	Ref	**3.07 (1.03, 5.11)**	**3.51 (1.35, 5.67)**	0.69 (−1.5, 2.88)	0.58 (−1.24, 2.4)
Diabetes					
Yes	Ref	−1.34 (−4.29, 1.61)	1.25 (−2.06, 4.56)	−0.88 (−4.14, 2.39)	1.28 (−1.52, 4.09)
No	Ref	**1.57 (0.26, 2.87)**	**1.83 (0.44, 3.23)**	0.63 (−0.58, 1.83)	**1.57 (0.59, 2.55)**
Treatment for hypertension					
Yes	Ref	**2.27 (0.67, 3.88)**	**2.90 (1.11, 4.69)**	0.25 (−1.38, 1.88)	0.50 (−0.83, 1.82)
No	Ref	1.41 (−3.43, 6.24)	**5.01 (0.88, 9.13)**	0.48 (−2.93, 3.88)	0.46 (−2.62, 3.55)
Smoker					
Yes	Ref	1.20 (−1.76, 4.16)	**3.55 (0.07, 7.04)**	1.12 (−2.55, 4.78)	0.18 (−2.70, 3.06)
No	Ref	**1.73 (0.06, 3.40)**	**1.92 (0.18, 3.66)**	0.26 (−1.23, 1.74)	−0.11 (−1.35, 1.13)

Values are β (95% confidence interval). Adjusted for sex, race/ethnicity, BMI, income-poverty ratio, education level, marital status, and physical activity. The model is not adjusted for the stratification variable itself. Boldface indicates statistical significance (*p* < 0.05). BMI, body mass index; ASCVD, atherosclerotic cardiovascular disease.

## 4. Discussion

The present study used a nationally representative sample of community-dwelling adults aged 60–79 years and assessed the risk of a first hard ASCVD event in the next 10 years according to the 2013 ACC/AHA equation. In this study, we found an inverse association between weight loss ≥5% and the predicted 10-year ASCVD risk in older adults, and no protective effect of weight gain.

Our results are generally in accordance with previous studies on the relationship between weight loss and cardiovascular and all-cause mortality in older adults. In a longitudinal observational cohort study conducted in the U.S. communities, weight loss of 5% or more in 3 years was associated with an increased risk of mortality in older adults (8). Likewise, community-dwelling older Japanese people with weight loss ≥5% in all terms was associated with a higher risk of all-cause mortality, not only in short-term (3 years), but also in medium-term (6–7 years) and long-term (12–13 years) (14). Moreover, a Tehran study of participants with type 2 diabetes aged ≥60 years without a history of cardiovascular disease and cancer at baseline has suggested that 3-year weight loss >5% was associated with an increased risk (marginally significant) of incident cardiovascular disease during more than 14 years of follow-up (15).

However, some previous studies reported conflicting results. For example, in a 12-month randomized controlled trial (RCT) of 164 obese older adults aged ≥65 years, there was a positive significant association between weight loss by exercise plus moderate caloric restriction and cardiometabolic risk (16). In addition, another RCT comprising 585 obese participants aged 60–80 years with hypertension found a favorable association between intentional weight loss (mean 4.4 kg) and reductions in the need for antihypertensive medication (17). This study also reported weight loss was not significantly associated with increased cardiovascular disease events during the first 2 years of follow-up (17), nor was all-cause mortality during over 12 years of follow-up (18).

In addition to the weight change calculated in different time intervals, the discrepancy between our study and previous ones may partly be related to current BMI and intention to lose weight. Indeed, our study indicated that the association between weight loss and the predicted 10-year risk was not significant in obesity (≥ 5 kg/m^2^) and intentional weight loss. This may be due to the fact that unintentional weight loss in older adults is usually reflect disease severity (e.g., in patients with advanced heart disease, lung disease or malignant disease) or underlying disease, and is particularly relevant to worsened outcomes (19). Cancer or malignancy, which is characterized by substantial weight loss during 1 year or less, accounts for 16–36% of organic causes of unintentional weight loss in older adults (20). However, our study showed that, an increased risk of a first ASCVD event was associated with weight loss within 10 years rather than 1 year. In addition, another possibility for discrepancy is the difference in the level of physical activity. The loss of muscle mass (21) and bone strength (22) that occurs with age, which contributes to disability and frailty in older adults (23, 24). Physical activity alone, without diet control, is not typically associated with significant weight loss and it can maintain lean body mass and muscle strength, improve cardiometabolic risk factors and physical function in older adults (7, 25, 26). However, because of the opposite effect observed in our study between vigorous and moderate intensity activity, further research is needed to clarify this issue.

A major strength of our study is the use of a nationally representative sample that represents the general U.S. adult population. Furthermore, with the comprehensive data collected in NHANES, a wide range of potential confounders including demographic, socioeconomic and lifestyle were able to be controlled.

As for the limitations. First, we used self-reported weight for the analysis, instead of measured weight, which may lead to the misclassification of weight change status. Secondly, we did not consider the fluctuation of body weight during the 10-year intervals. Third, weight lacks discriminatory power to differentiate between body fat and lean mass. Therefore, future research should investigate the same question using indicators of body composition to more fully understand the mechanisms linking weight changes to ASCVD risk to mortality risk. Fourthly, we failed to consider specific modalities of losing weight, such as intermittent fasting, low-calorie diets, more exercise or bariatric surgery, which might cause bias. Finally, this study was cross-sectional research, and it cannot demonstrate the causation but only the association. Further and prospective studies should be completed in the future.

## 5. Conclusion

In this study of U.S. adults 60–79 years of age, we found that weight loss >5% over 10 years was significantly associated with an increased the predicted 10-year ASCVD risk. Our study supports the benefits of stable weight in promoting cardiovascular health in older adults. However, further prospective studies are required to elucidate the effect of weight management in later life on decreasing ASCVD risk.

## Data availability statement

The datasets presented in this study can be found in online repositories. The names of the repository/repositories and accession number(s) can be found in the article/Supplementary material.

## Ethics statement

The NHANES study was approved by the National Center for Health Statistics’ Ethics Review Board. The study was conducted in accordance with the local legislation and institutional requirements. All participants provided written informed consent (parental consent was obtained for those < 18 years).

## Author contributions

YP: full access to all of the data in the study and takes responsibility for the integrity of the data and the accuracy of the data analysis. YP and HL: concept and design. JL, FL, and WY: acquisition, analysis, and interpretation of data. LT, AL, and YW: drafting of the manuscript. YP and HQ: statistical analysis. LL and HQ: administrative, technical, and material support. CF: supervision. All authors contributed to the article and approved the submitted version.

## Footnotes

^1^http://www.R-project.org

^2^www.empowerstats.com

## Abbreviations

ASCVD: Atherosclerotic cardiovascular disease
NHANES: National Health and Nutrition Examination Survey
NCHS: National Center for Health Statistics
HDL-C: High-density lipoprotein cholesterol
TC: Total cholesterol
MI: Myocardial infarction
CHD: Coronary heart disease
PCEs: Pooled cohort equations
ACC: American College of Cardiology
AHA: American Heart Association
BMI: Body mass index
SBP: Systolic blood pressure
DBP: Diastolic blood pressure
CI: Confidence interval
RCT: Randomized controlled trial

## References

[1] Afshin A Forouzanfar MH Reitsma MB Sur P Estep K Lee A. Health effects of overweight and obesity in 195 countries over 25 years. N Engl J Med. (2017) 377:13–27. doi: 10.1056/NEJMoa1614362, PMID: 28604169 PMC5477817

[2] Hales CM Fryar CD Carroll MD Freedman DS Ogden CL. Trends in obesity and severe obesity prevalence in US youth and adults by sex and age, 2007-2008 to 2015-2016. JAMA. (2018) 319:1723–5. doi: 10.1001/jama.2018.3060, PMID: 29570750 PMC5876828

[3] Stevens J Cai J Pamuk ER Williamson DF Thun MJ Wood JL. The effect of age on the association between body-mass index and mortality. N Engl J Med. (1998) 338:1–7. doi: 10.1056/NEJM199801013380101 9414324

[4] Angelantonio E Bhupathiraju S Wormser D Gao P Kaptoge S de Gonzalez AB. Body-mass index and all-cause mortality: individual-participant-data meta-analysis of 239 prospective studies in four continents. Lancet. (2016) 388:776–86. doi: 10.1016/S0140-6736(16)30175-1, PMID: 27423262 PMC4995441

[5] Jensen MD Ryan DH Apovian CM Ard JD Comuzzie AG Donato KA. 2013 AHA/ACC/TOS guideline for the management of overweight and obesity in adults: a report of the American College of Cardiology/American Heart Association task force on practice guidelines and the Obesity Society. Circulation. (2014) 129:S102–38. doi: 10.1161/01.cir.0000437739.71477.ee, PMID: 24222017 PMC5819889

[6] Villareal DT Aguirre L Gurney AB Waters DL Sinacore DR Colombo E. Aerobic or resistance exercise, or both, in dieting obese older adults. N Engl J Med. (2017) 376:1943–55. doi: 10.1056/NEJMoa1616338, PMID: 28514618 PMC5552187

[7] Batsis JA Gill LE Masutani RK Adachi-Mejia AM Blunt HB Bagley PJ. Weight loss interventions in older adults with obesity: a systematic review of randomized controlled trials since 2005. J Am Geriatr Soc. (2017) 65:257–68. doi: 10.1111/jgs.14514, PMID: 27641543 PMC5414418

[8] Newman AB Yanez D Harris T Duxbury A Enright PL Fried LP. Weight change in old age and its association with mortality. J Am Geriatr Soc. (2001) 49:1309–18. doi: 10.1046/j.1532-5415.2001.49258.x 11890489

[9] Alharbi TA Paudel S Gasevic D Ryan J Freak-Poli R Owen AJ. The association of weight change and all-cause mortality in older adults: a systematic review and meta-analysis. Age Ageing. (2021) 50:697–704. doi: 10.1093/ageing/afaa231, PMID: 33161429

[10] Romero-Corral A Montori VM Somers VK Korinek J Thomas RJ Allison TG. Association of bodyweight with total mortality and with cardiovascular events in coronary artery disease: a systematic review of cohort studies. Lancet. (2006) 368:666–78. doi: 10.1016/S0140-6736(06)69251-9, PMID: 16920472

[11] Pack QR Rodriguez-Escudero JP Thomas RJ Ades PA West CP Somers VK. The prognostic importance of weight loss in coronary artery disease: a systematic review and meta-analysis. Mayo Clin Proc. (2014) 89:1368–77. doi: 10.1016/j.mayocp.2014.04.033 25199859 PMC4734114

[12] World Health Organization. Technical advisory group for measurement, monitoring and evaluation of the UN decade of healthy ageing. Available at: https://www.who.int/groups/technical-advisory-group-for-measurement-monitoring-and-evaluation-of-the-un-decade-of-healthy-ageing (2022).

[13] Goff DCJ Lloyd-Jones DM Bennett G Coady S D’Agostino RB Gibbons R. 2013 ACC/AHA guideline on the assessment of cardiovascular risk: a report of the American College of Cardiology/American Heart Association task force on practice guidelines. Circulation. (2014) 129:S49–73. doi: 10.1161/01.cir.0000437741.48606.98, PMID: 24222018

[14] Murayama H Liang J Shaw BA Botoseneanu A Kobayashi E Fukaya T. Short-, medium-, and long-term weight changes and all-cause mortality in old age: findings from the National Survey of the Japanese elderly. J Gerontol A Biol Sci Med Sci. (2021) 76:2039–46. doi: 10.1093/gerona/glab052, PMID: 33626135 PMC8514062

[15] Moazzeni SS Hizomi Arani R Deravi N Hasheminia M Khalili D Azizi F. Weight change and risk of cardiovascular disease among adults with type 2 diabetes: more than 14 years of follow-up in the Tehran lipid and glucose study. Cardiovasc Diabetol. (2021) 20:141. doi: 10.1186/s12933-021-01326-2 34253199 PMC8276460

[16] Ard JD Gower B Hunter G Ritchie CS Roth DL Goss A. Effects of calorie restriction in obese older adults: the CROSSROADS randomized controlled trial. J Gerontol A Biol Sci Med Sci. (2017) 73:glw237–80. doi: 10.1093/gerona/glw237, PMID: 28003374 PMC5861948

[17] Whelton PK Appel LJ Espeland MA Applegate WB Ettinger WHJ Kostis JB. Sodium reduction and weight loss in the treatment of hypertension in older persons: a randomized controlled trial of nonpharmacologic interventions in the elderly (TONE). TONE Collaborative Research Group. JAMA. (1998) 279:839–46. doi: 10.1001/jama.279.11.839, PMID: 9515998

[18] Brown RE Kuk JL. Consequences of obesity and weight loss: a devil's advocate position. Obes Rev. (2015) 16:77–87. doi: 10.1111/obr.12232, PMID: 25410935 PMC4312481

[19] Alibhai SM Greenwood C Payette H. An approach to the management of unintentional weight loss in elderly people. CMAJ. (2005) 172:773–80. doi: 10.1503/cmaj.1031527, PMID: 15767612 PMC552892

[20] McMinn J Steel C Bowman A. Investigation and management of unintentional weight loss in older adults. BMJ. (2011) 342:d1732. doi: 10.1136/bmj.d1732 21447571

[21] Newman AB Lee JS Visser M Goodpaster BH Kritchevsky SB Tylavsky FA. Weight change and the conservation of lean mass in old age: the health, aging and body composition study. Am J Clin Nutr. (2005) 82:872–8. doi: 10.1093/ajcn/82.4.872, PMID: 16210719

[22] Ensrud KE Vo TN Burghardt AJ Schousboe JT Cauley JA Taylor BC. Weight loss in men in late life and bone strength and microarchitecture: a prospective study. Osteoporos Int. (2018) 29:1549–58. doi: 10.1007/s00198-018-4489-6, PMID: 29572622 PMC6035779

[23] Gill LE Bartels SJ Batsis JA. Weight Management in Older Adults. Curr Obes Rep. (2015) 4:379–88. doi: 10.1007/s13679-015-0161-z, PMID: 26627496 PMC5387759

[24] Grabowski DC Ellis JE. High body mass index does not predict mortality in older people: analysis of the longitudinal study of aging. J Am Geriatr Soc. (2001) 49:968–79. doi: 10.1046/j.1532-5415.2001.49189.x 11527490

[25] Brennan AM Standley RA Anthony SJ Grench KE Helbling NL DeLany JP. Weight loss and exercise differentially affect insulin sensitivity, body composition, cardiorespiratory fitness, and muscle strength in older adults with obesity: a randomized controlled trial. J Gerontol A Biol Sci Med Sci. (2022) 77:1088–97. doi: 10.1093/gerona/glab240, PMID: 34406407 PMC9071425

[26] Bouchonville M Armamento-Villareal R Shah K Napoli N Sinacore DR Qualls C. Weight loss, exercise or both and cardiometabolic risk factors in obese older adults: results of a randomized controlled trial. Int J Obes. (2014) 38:423–31. doi: 10.1038/ijo.2013.122, PMID: 23823329 PMC3835728

