# Supplementary material for: Association between weight change and the predicted 10-year risk for atherosclerosis cardiovascular disease among U.S. older adults: data from National Health and Nutrition Examination Survey 1999–2018
Source: Front Public Health. 2023 Oct 16;11:1183200. doi: 10.3389/fpubh.2023.1183200 (PMC10614635; doi:10.3389/fpubh.2023.1183200)
Supplement: Supplementary file 1 [file Table_1.DOCX]

Supplementary Material 1: Characteristics of study participants according to 1-year weight change patterns in National Health and Nutrition Examination survey, 1999–2018.

| **Weight change** | **All participants**  **(n=1,894)** | **Weight stable**  **(n=1,034)** | **Moderate-to-large weight loss**  **(n=193)** | **Small weight loss (n=294)** | **Small weight gain**  **(n=205)** | **Moderate-to-large weight gain**  **(n=168)** | **P-value** |
| --- | --- | --- | --- | --- | --- | --- | --- |
| **Age (years),** **mean (SD)** | 67.49 ± 5.37 | 67.58 ± 5.40 | 67.93 ± 5.43 | 67.66 ± 5.37 | 66.88 ± 5.30 | 66.90 ± 5.09 | 0.16 |
| **Sex, n (%)** |  |  |  |  |  |  | **<0.001** |
| Male | 1092 (57.66%) | 641 (61.99%) | 113 (58.55%) | 183 (62.24%) | 93 (45.37%) | 62 (36.90%) |  |
| Female | 802 (42.34%) | 393 (38.01%) | 80 (41.45%) | 111 (37.76%) | 112 (54.63%) | 106 (63.10%) |  |
| **Race/ethnicity, n (%)** |  |  |  |  |  |  | **<0.001** |
| Mexican American | 295 (15.58%) | 157 (15.18%) | 30 (15.54%) | 46 (15.65%) | 39 (19.02%) | 23 (13.69%) |  |
| Other Hispanic | 155 (8.18%) | 94 (9.09%) | 18 (9.33%) | 25 (8.50%) | 8 (3.90%) | 10 (5.95%) |  |
| Non-Hispanic white | 815 (43.03%) | 474 (45.84%) | 79 (40.93%) | 98 (33.33%) | 93 (45.37%) | 71 (42.26%) |  |
| Non-Hispanic Black | 539 (28.46%) | 252 (24.37%) | 60 (31.09%) | 114 (38.78%) | 56 (27.32%) | 57 (33.93%) |  |
| Other race | 90 (4.75%) | 57 (5.51%) | 6 (3.11%) | 11 (3.74%) | 9 (4.39%) | 7 (4.17%) |  |
| **BMI (kg/m^2^),** **mean ± SD** | 30.22 ± 6.14 | 29.92 ± 5.71 | 28.37 ± 5.92 | 29.67 ± 6.46 | 31.63 ± 6.19 | 33.41 ± 6.87 | **<0.001** |
| **Income poverty ratio,** **mean ± SD** | 2.50 ± 1.58 | 2.68 ± 1.61 | 2.05 ± 1.42 | 2.32 ± 1.51 | 2.51 ± 1.54 | 2.20 ± 1.49 | **<0.001** |
| **Educational level, n (%)** |  |  |  |  |  |  | **0.004** |
| Lower | 313 (16.55%) | 162 (15.68%) | 48 (25.13%) | 54 (18.37%) | 28 (13.66%) | 21 (12.50%) |  |
| Intermediate | 1262 (66.74%) | 679 (65.73%) | 125 (65.45%) | 196 (66.67%) | 141 (68.78%) | 121 (72.02%) |  |
| Higher | 316 (16.71%) | 192 (18.59%) | 18 (9.42%) | 44 (14.97%) | 36 (17.56%) | 26 (15.48%) |  |
| **Marital status, n (%)** |  |  |  |  |  |  | **<0.001** |
| Married/partnered | 1152 (61.28%) | 677 (66.24%) | 103 (53.65%) | 167 (57.00%) | 111 (54.15%) | 94 (55.95%) |  |
| Single/no partner | 728 (38.72%) | 345 (33.76%) | 89 (46.35%) | 126 (43.00%) | 94 (45.85%) | 74 (44.05%) |  |
| **Physical activity, n (%)** |  |  |  |  |  |  | **0.016** |
| Vigorous activity | 205 (10.82%) | 126 (12.19%) | 14 (7.25%) | 32 (10.88%) | 21 (10.24%) | 12 (7.14%) |  |
| Moderate activity | 559 (29.51%) | 323 (31.24%) | 48 (24.87%) | 82 (27.89%) | 67 (32.68%) | 39 (23.21%) |  |
| No | 1130 (59.66%) | 585 (56.58%) | 131 (67.88%) | 180 (61.22%) | 117 (57.07%) | 117 (69.64%) |  |
| **Smoker, n (%)** |  |  |  |  |  |  | **0.002** |
| Yes | 489 (25.82%) | 238 (23.02%) | 68 (35.23%) | 89 (30.27%) | 54 (26.34%) | 40 (23.81%) |  |
| No | 1405 (74.18%) | 796 (76.98%) | 125 (64.77%) | 205 (69.73%) | 151 (73.66%) | 128 (76.19%) |  |
| **Diabetes, n (%)** |  |  |  |  |  |  | 0.051 |
| Yes | 529 (27.93%) | 268 (25.92%) | 69 (35.75%) | 88 (29.93%) | 53 (25.85%) | 51 (30.36%) |  |
| No | 1365 (72.07%) | 766 (74.08%) | 124 (64.25%) | 206 (70.07%) | 152 (74.15%) | 117 (69.64%) |  |
| **Treatment for hypertension, n (%)** |  |  |  |  |  |  | 0.692 |
| Yes | 1733 (91.50%) | 941 (91.01%) | 177 (91.71%) | 274 (93.20%) | 185 (90.24%) | 156 (92.86%) |  |
| No | 161 (8.50%) | 93 (8.99%) | 16 (8.29%) | 20 (6.80%) | 20 (9.76%) | 12 (7.14%) |  |
| **SBP (mmHg),** **mean ± SD** | 136.21 ± 19.63 | 136.03 ± 19.50 | 134.76 ± 22.56 | 136.73 ± 19.32 | 135.60 ± 19.57 | 138.78 ± 17.27 | 0.352 |
| **DBP (mmHg),** **mean ± SD** | 70.55 ± 12.35 | 70.37 ± 12.36 | 68.47 ± 11.74 | 71.30 ± 12.74 | 71.16 ± 12.03 | 72.02 ± 12.42 | **0.047** |
| **TC (mg/dL),** **mean ± SD** | 196.17 ± 36.70 | 196.33 ± 36.16 | 189.97 ± 35.52 | 195.24 ± 38.63 | 197.68 ± 36.95 | 202.08 ± 36.88 | **0.034** |
| **HDL (mg/dL),** **mean ± SD** | 52.49 ± 15.04 | 51.82 ± 15.03 | 55.10 ± 15.85 | 52.67 ± 15.27 | 51.77 ± 14.60 | 54.20 ± 14.01 | **0.032** |
| **10-year ASCVD risk，mean ± SD** | 23.24 ± 13.14 | 23.10 ± 12.90 | 24.94 ± 14.18 | 24.64 ± 12.81 | 21.28 ± 13.13 | 22.15 ± 13.64 | **0.015** |

BMI, body mass index; SBP, systolic blood pressure; DBP, diastolic blood pressure; TC, total cholesterol; HDL-C, high-density lipoprotein cholesterol; ASCVD, atherosclerotic cardiovascular disease; SD, standard deviation

Supplementary Material 2: Association between 1-year weight change and 10-year ASCVD risk.

|  |  | **Model 1** | | **Model 2** | | **Model3** | |
| --- | --- | --- | --- | --- | --- | --- | --- |
|  | N | **β (95% CI)** | **P** | **β (95% CI)** | **P** | **β (95% CI)** | **P** |
| **Weight change** | 1,894 | **-0.12 (-0.18, -0.06)** | **＜0.01** | -0.01 (-0.06, 0.04) | 0.82 | 0.01(-0.04,0.06) | 0.70 |
| **Weight change pattern** |  |  |  |  |  |  |  |
| Loss ≥10% | 193 (10.19%) | 1.84 (-0.18, 3.85) | 0.07 | 1.37 (-0.20, 2.93) | 0.08 | 0.50(-1.06,2.06) | 0.53 |
| Loss -5.1%~-9.9% | 294 (15.52%) | 1.54 (-0.16, 3.24) | 0.07 | 0.73 (-0.60, 2.06) | 0.28 | 0.34(-0.97,1.65) | 0.61 |
| Stable (±5.0%) | 1,034 (54.59%) | Ref | | Ref | | Ref | |
| Gain 5.1%~9.9% | 205 (10.82%) | -1.82 (-3.78, 0.15) | 0.07 | 0.28 (-1.26, 1.82) | 0.72 | 0.17(-1.35,1.68) | 0.83 |
| Gain ≥10% | 168 (8.87%) | -0.95 (-3.08, 1.19) | 0.38 | 1.60 (-0.08, 3.28) | 0.06 | 1.29(-0.39,2.96) | 0.13 |

Model 1, no covariates were adjusted;

Model 2, age, sex, and race/ethnicity were adjusted;

Model 3, age, sex, race/ethnicity, body mass index, income-poverty ratio, physical activity, education level, and marital status were adjusted.

ASCVD, atherosclerotic cardiovascular disease; CI, confidence interval

Supplementary Material 3: Stratified analyses of the association between 1-year weight change and 10-year ASCVD risk.

|  | **Weight stable** | | |  | **Moderate-to-large weight loss** |  | **Small weight loss** |  | **Small weight gain** |  | **Moderate-to-large weight gain** |
| --- | --- | --- | --- | --- | --- | --- | --- | --- | --- | --- | --- |
| **Age** |  |  | |  |  |  |  |  |  |  |  |
| 60-64 | Ref | | |  | 0.40(-1.85,2.64) |  | -0.23(-2.08,1.62) |  | -1.18(-3.12,0.76) |  | -0.34(-2.49,1.81) |
| 65-69 | Ref | | |  | 2.77(-0.07,5.61) |  | 1.30(-0.99,3.59) |  | 1.77(-1.08,4.61) |  | 1.18(-1.87,4.23) |
| 70-74 | Ref | | |  | -1.21(-4.49,2.07) |  | 0.73(-2.25,3.71) |  | 1.59(-1.95,5.14) |  | **4.07(0.20,7.95)** |
| 75-79 | Ref | | |  | 0.77(-5.04,6.58) |  | -2.06(-6.9,2.79) |  | -3.58(-9.86,2.7) |  | 3.52(-3.95,10.99) |
| **Sex** |  | |  |  |  |  |  |  |  |  |  |
| Male | Ref | | |  | -0.50(-2.58,1.59) |  | 0.81(-0.90,2.52) |  | 1.21(-1.05,3.47) |  | 0.91(-1.80,3.62) |
| Female | Ref | | |  | 1.91(-0.45,4.27) |  | -0.30(-2.37,1.78) |  | -0.85(-2.91,1.20) |  | 1.52(-0.61,3.65) |
| **Race/ethnicity** |  | |  |  |  |  |  |  |  |  |  |
| Mexican American | Ref | | |  | -1.85(-5.43,1.72) |  | -0.38(-3.41,2.65) |  | 0.80(-2.46,4.07) |  | -0.37(-4.49,3.75) |
| Other Hispanic | Ref | | |  | -1.61(-6.34,3.13) |  | 0.62(-3.46,4.69) |  | 0.19(-6.66,7.03) |  | -1.31(-6.98,4.37) |
| Non-Hispanic white | Ref | | |  | 1.55(-0.53,3.63) |  | 0.15(-1.73,2.03) |  | 0.25(-1.69,2.19) |  | 0.98(-1.23,3.20) |
| Non-Hispanic Black | Ref | | |  | 0.66(-2.90,4.22) |  | 0.33(-2.40,3.06) |  | 0.14(-3.4,3.68) |  | 2.76(-0.83,6.35) |
| Other race | Ref | | |  | **10.73(1.17,20.29)** |  | 1.57(-5.85,9.00) |  | -3.40(-11.25,4.46) |  | 1.16(-8.51,10.83) |
| **BMI** |  | | |  |  |  |  |  |  |  |  |
| ＜25.0 | Ref | | |  | 1.44(-2.46,5.33) |  | 0.67(-2.94,4.27) |  | -4.91(-10.96,1.14) |  | -1.88(-8.69,4.93) |
| 25.0-29.9 | Ref | | |  | -0.92(-3.33,1.49) |  | 0.51(-1.49,2.51) |  | 2.07(-0.36,4.51) |  | -0.25(-3.38,2.88) |
| 30.0-34.9 | Ref | | |  | 0.38(-3.01,3.76) |  | 0.02(-2.65,2.69) |  | -1.68(-4.30,0.93) |  | 0.91(-1.96,3.79) |
| ≥35.0 | Ref | | |  | 1.48(-2.44,5.39) |  | 0.35(-2.79,3.50) |  | 2.73(-0.61,6.08) |  | **3.20(0.24,6.16)** |
| **Intention to lose weight** |  | | |  |  |  |  |  |  |  |  |
| yes | Ref | | |  | -1.67(-4.65,1.31) |  | 0.01(-2.68,2.70) |  | 1.75(-1.51,5.01) |  | 1.15(-2.59,4.9) |
| no | Ref | | |  | 0.62(-1.04,2.29) |  | 0.18(-1.17,1.54) |  | -0.74(-2.28,0.8) |  | 1.24(-0.43,2.92) |
| **Physical activity** |  | | |  |  |  |  |  |  |  |  |
| Vigorous activity | Ref | | |  | 3.54(-1.77,8.85) |  | 2.52(-1.37,6.42) |  | -0.70(-5.10,3.70) |  | 5.17(-1.04,11.39) |
| Moderate activity | Ref | | |  | -0.45(-3.42,2.52) |  | -0.92(-3.28,1.45) |  | -2.20(-4.81,0.40) |  | -0.50(-3.83,2.83) |
| No | Ref | | |  | 0.72(-1.30,2.75) |  | 0.49(-1.28,2.27) |  | 1.39(-0.73,3.51) |  | 1.72(-0.41,3.86) |
| **Treatment for hypertension** |  | | |  |  |  |  |  |  |  |  |
| Yes | Ref | | |  | -0.19(-6.00,5.62) |  | -0.03(-3.20,3.14) |  | -0.39(-2.57,1.78) |  | 1.28(-1.10,3.65) |
| No | Ref | | |  | 1.52(-0.89,3.94) |  | 0.24(-1.73,2.21) |  | 0.03(-2.12,2.19) |  | 1.28(-1.12,3.68) |
| **Diabetes** |  | | |  |  |  |  |  |  |  |  |
| Yes | Ref | | |  | -0.56(-3.43,2.30) |  | -0.18(-2.82,2.47) |  | 1.63(-1.52,4.78) |  | 2.84(-0.49,6.17) |
| No | Ref | | |  | -0.81(-2.11,0.50) |  | 0.20(-0.85,1.25) |  | -0.03(-1.22,1.16) |  | 0.62(-0.73,1.97) |
| **Smoker** |  | | |  |  |  |  |  |  |  |  |
| Yes | Ref | | |  | 0.59(-1.06,2.23) |  | 0.25(-1.12,1.63) |  | 0.20(-1.41,1.81) |  | 1.47(-0.29,3.23) |
| No | Ref | | |  | 0.17(-4.05,4.40) |  | 3.18(-0.80,7.16) |  | 0.63(-3.20,4.46) |  | 0.61(-4.03,5.25) |

Values are β (95% confidence interval). Adjusted for sex, race/ethnicity, BMI, income-poverty ratio, education level, marital status, and physical activity. The model is not adjusted for the stratification variable itself. Boldface indicates statistical significance (p<0.05).

BMI, body mass index; ASCVD, atherosclerotic cardiovascular disease
